# Large Scale Screening of Southern African Plant Extracts for the Green Synthesis of Gold Nanoparticles Using Microtitre-Plate Method

**DOI:** 10.3390/molecules21111498

**Published:** 2016-11-08

**Authors:** Abdulrahman M. Elbagory, Christopher N. Cupido, Mervin Meyer, Ahmed A. Hussein

**Affiliations:** 1 DST/Mintek Nanotechnology Innovation Centre, Department of Biotechnology, University of the Western Cape, Private Bag X17, Bellville 7530, South Africa; 3376881@myuwc.ac.za (A.M.E.); memeyer@uwc.ac.za (M.M.); 2 South African National Biodiversity Institute, Compton Herbarium, Private bag X7, Claremont 7735, South Africa; C.Cupido@sanbi.org.za; 3 Department of Biodiversity and Conservation Biology, University of the Western Cape, Private Bag X17, Bellville 7535, South Africa; 4 Department of Chemistry, University of the Western Cape, Private Bag X17, Bellville 7530, South Africa

**Keywords:** green nanotechnology, gold nanoparticles, biosynthesis, high resolution transmission electron microscopy, Cape flora

## Abstract

The preparation of gold nanoparticles (AuNPs) involves a variety of chemical and physical methods. These methods use toxic and environmentally harmful chemicals. Consequently, the synthesis of AuNPs using green chemistry has been under investigation to develop eco-friendly nanoparticles. One approach to achieve this is the use of plant-derived phytochemicals that are capable of reducing gold ions to produce AuNPs. The aim of this study was to implement a facile microtitre-plate method to screen a large number of aqueous plant extracts to determine the optimum concentration (OC) for the bio-synthesis of the AuNPs. Several AuNPs of different sizes and shapes were successfully synthesized and characterized from 17 South African plants. The characterization was done using Ultra Violet-Visible Spectroscopy, Dynamic Light Scattering, High Resolution Transmission Electron Microscopy and Energy-Dispersive X-ray Spectroscopy. We also studied the effects of temperature on the synthesis of the AuNPs and showed that changes in temperatures affect the size and dispersity of the generated AuNPs. We also evaluated the stability of the synthesized AuNPs and showed that some of them are stable in biological buffer solutions.

## 1. Introduction

Metallic nanoparticles have potential applications in chemistry, physics and biology due to their unequalled optical, electrical and photothermal properties [1]. These metal nanoparticles have drawn researchers’ attention because of the ease of their synthesis and modification [2]. Among the metal nanoparticles, gold nanoparticles (AuNPs) have received much attention for their unique and adjustable Surface Plasmon Resonance (SPR) [3]. AuNPs have been utilized in several biomedical applications such as drug delivery, disease diagnosis, treatment of cancer, photothermal therapy and immunochromatographic identification of pathogens in clinical specimens [4,5,6,7].

In general, the preparation of metal nanoparticles involves a variety of chemical and physical methods, such as chemical reduction [8], photochemical reduction [9], electrochemical reduction [10], laser ablation [11] and lithography [12]. These methods are expensive and involve the use of several toxic, environmentally harmful inorganic chemicals, such as sodium/potassium borohydrate, hydrazine and salts of tartrate, or organic chemicals, such as sodium citrate, ascorbic acid and amino acids, which are used for their reducing capabilities [13]. The employment of these harmful chemicals can limit the use of nanoparticles in biomedical applications [14].

Consequently, the green synthesis of AuNPs has been under investigation owing to the rising need to develop biocompatible, less-toxic and eco-friendly nanoparticles. One method to achieve this is the utilization of biological systems such as bacteria, fungi and plant extracts. For example, Kalishwaralal and co-workers synthesized gold nanocubes, ranging from 10 to 100 nm, from the bacterium *Bacillus licheniformis* after incubation with gold salt for 48 h [15]. Shankar et al., synthesized spherical AuNPs from an endophytic fungus (*Colletotrichum* sp.) after 96 h of incubation [16]. Several studies reported the synthesis of AuNPs using extracts from plants such as, *Aloe vera* [17], *Terminalia catappa* [18], *Suaeda monoica* [19], *Trianthema decandra* [1] and *Memecylon umbellatum* [20]. These synthesis methods are not only eco-friendly, but also cost-effective and can be easily modified for large-scale synthesis [1].

The use of plants is more attractive, compared to the other biological systems, as they are readily available, safer and contain wide variety of reducing phytochemicals. Compared to microbial-derived chemicals, the plant-derived phytochemicals require shorter incubation periods with gold salt in order to synthesize AuNPs [2]. These phytochemicals are not only responsible for the synthesis of metal nanoparticles, but also they act as capping agents to prevent the coalescence of colloidal particles, which are kept apart in solution by electrostatic forces [13]. It is thought that different-shaped polyol and water-soluble heterocyclic components of plant phytochemicals are mainly responsible for the reduction and coating the gold ions [20].

The flora of the South Western Cape, which is commonly referred to as the Cape Flora or the Core Cape Sub-region of the Greater Cape Floristic Region, is the smallest and richest floral kingdom in the world. It has over 9300 species occupying a land area of approximately 90,000 km^2^ with about 70% of the species occurring nowhere else in the world [21].

Herein we report the synthesis of AuNPs from aqueous extracts prepared from 17 plants collected from the South Western Cape area of South Africa. The synthesis process was monitored under two different temperature conditions to measure the effect of temperature on the geometric properties of the synthesized AuNPs. The stability of the AuNPs was measured in different biological buffer solutions. Several physical and optical measurement techniques including, Ultraviolet-Visible Spectroscopy (UV-Vis), Dynamic Light Scattering (DLS), High Resolution Transmission Electron Microscopy (HR-TEM) and Energy-dispersive X-ray spectroscopy (EDS) were used to characterize the AuNPs.

## 2. Results and Discussion

Previous studies reported the green synthesis of the plant-extract mediated AuNPs by mixing fixed concentrations of gold salt solutions with the plant extracts solutions [1,17,18,19,20]. In this study, we sought to improve current methods used to biosynthesize AuNPs from plant extracts by developing micro-scale method to screen a large number of plants simultaneously. Using this method, we can also determine the optimum concentrations (OC) at which the plant extracts can reduce gold salts to form AuNPs.

### 2.1. Synthesis of AuNPs and Their UV-Vis Analysis

The formation of the AuNPs was visually observed by the development of red/wine-red colour in the 96 well plates. The measurement of the UV-Vis spectra also confirmed the formation of the AuNPs. A maxima absorbance between 500 and 600 nm (Table 1), is attributed to the excitation of AuNPs’ SPR [22] and considered as a distinct feature for the presence of AuNPs. Synthesis with three plant extracts namely; *Aspalathus hispida*, *Asparagus rubicundus*, and *Dicerothamnus rhinocertis*, did not produce any colour change at 25 °C. This may indicate the absence of strong reducing phytochemicals in their aqueous extracts, which may require higher temperatures to initiate the reduction process. However, since in this study the extracts were incubated with gold salt for 1 h, the synthesis of AuNPs with these plant extracts may also require longer periods to initiate the reduction of the gold ions at low temperature. Epigallocatechin gallate (EGCG), a phytochemical present in tea, was previously reported by Nune et al. to reduce gold salt [23] and used as a control to monitor the synthesize AuNPs. The λ_max_ of EGCG was 532 nm, which is within the same range of 530 nm the λ_max_ reported by Nune et al. [23].

**Figure 1:**
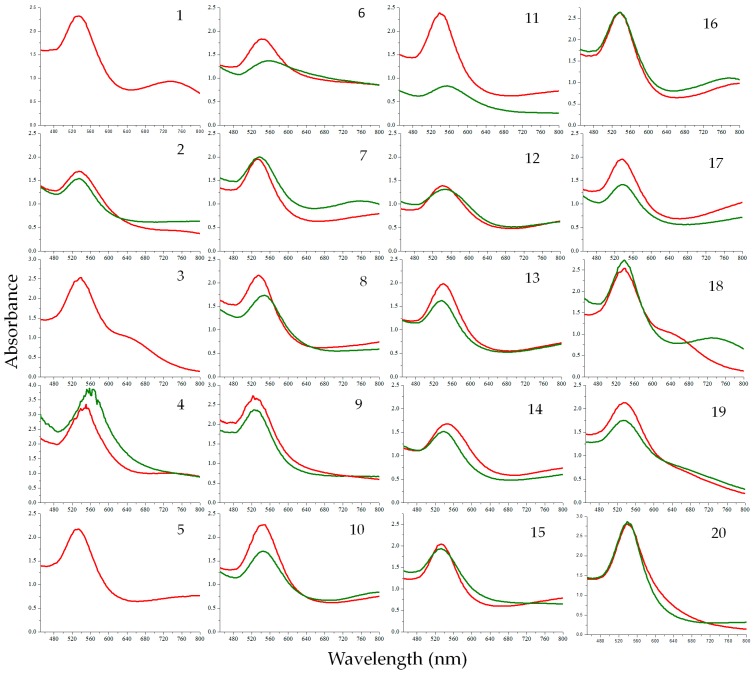
Comparison of the Ultraviolet-Visible Spectroscopy (UV-Vis) spectra for the AuNPs produced at 25 °C (**green line graph**) and at 70 °C (**red line graph**). The numbers on the spectra correspond to the numbers given to each plant in Table 1. *Camellia sinensis* (black tea) (18), *Camellia sinensis* (green tea) (19) and Epigallocatechin gallate (EGCG) (20) represent positive controls.

The SPR of the AuNPs can be affected by factors such as particle shape and size, the refractive index of the dispersion medium and the average distance between neighbouring AuNPs [24]. From the UV-Vis spectra of the AuNPs shown in Figure 1, it is evident that no major shifts were observed for AuNPs synthesized at 25 °C or at 70 °C. The notable difference observed, however, was the dissimilarity in the peaks’ height, which may relate to the number of the nanoparticles produced, as the OD-value correlates linearly with the concentration of the AuNPs in a solution [25]. Further, the bands generated by AuNPs synthesized at 70 °C were generally sharper and more symmetrical, which can be an indication of the increased uniformity in size distribution of AuNPs [26]. It was also observed that the plasmon bands of most of AuNPs are broad with an absorption tail in the longer wavelength attributing the excitation of the in-plane SPR and indicates significant anisotropy in the shape of gold nanoparticles [27] or the formation of aggregated spherical nanostructures [28]. For example, samples 1, 3, 16 and 18 (Figure 1) showed absorbance at higher wavelength, whereas green tea and EGCG (at both temperatures) showed a minimum absorption tail towards the near infrared region, which may indicate their stability and/or the lack of anisotropic nanoparticles compared to the previously mentioned samples. The relationship between the UV-Vis spectra and the polydispersity is also discussed later in Section 2.2.2. Table 1 summarize the maxima absorbance data recorded from all the plant extracts tested.

### 2.2. Particle Size Diameter, Distribution and Shape Analysis

#### 2.2.1. DLS Analysis

Synthesis at 25 °C produced nanoparticles with larger sizes for most of the plant extracts (Table 1). This is in agreement with previous studies, which also showed that the green synthesis of AuNPs at lower temperatures yielded larger AuNPs and that the synthesis of smaller nanoparticles can be optimised by increasing the temperature at which the synthesis is performed [29,30,31]. However, three of plant extracts (*E*. *africanus*, *H. alnifolia* and *I. brachystachya*) investigated in this study produced smaller AuNPs at 25 °C than at 70 °C. This may be due to the destruction of the capping agents in these plant extracts at high temperature which allowed the growth of larger particles. The smallest (47 nm) AuNPs produced at 25 °C was generated by the extract of *O. bracteolatum*, while the smallest (23 nm) AuNPs produced at 70 °C was obtained from green tea extract. The extract of *L. glaber* produced the largest AuNPs diameters of 218 nm and 136 nm at 25 °C and 70 °C, respectively.

The polydispersity index (Pdi) values in Table 1 also shows that the AuNPs are often more monodispersed when synthesized at 70 °C. The Pdi represents the ratio of particles of different size to total number of particles. A sample with low Pdi value more monodispersed. A study by Rajathi and co-workers, which reported the synthesis of AuNPs from the leaves of *S. monoica*, considered a sample with a Pdi value of 0.286 to be monodispersed particles [19]. AuNPs from *N. foetida* gave the lowest Pdi values both at 25 °C and 70 °C.

#### 2.2.2. HRTEM and EDX Analysis

**Figure 2:**
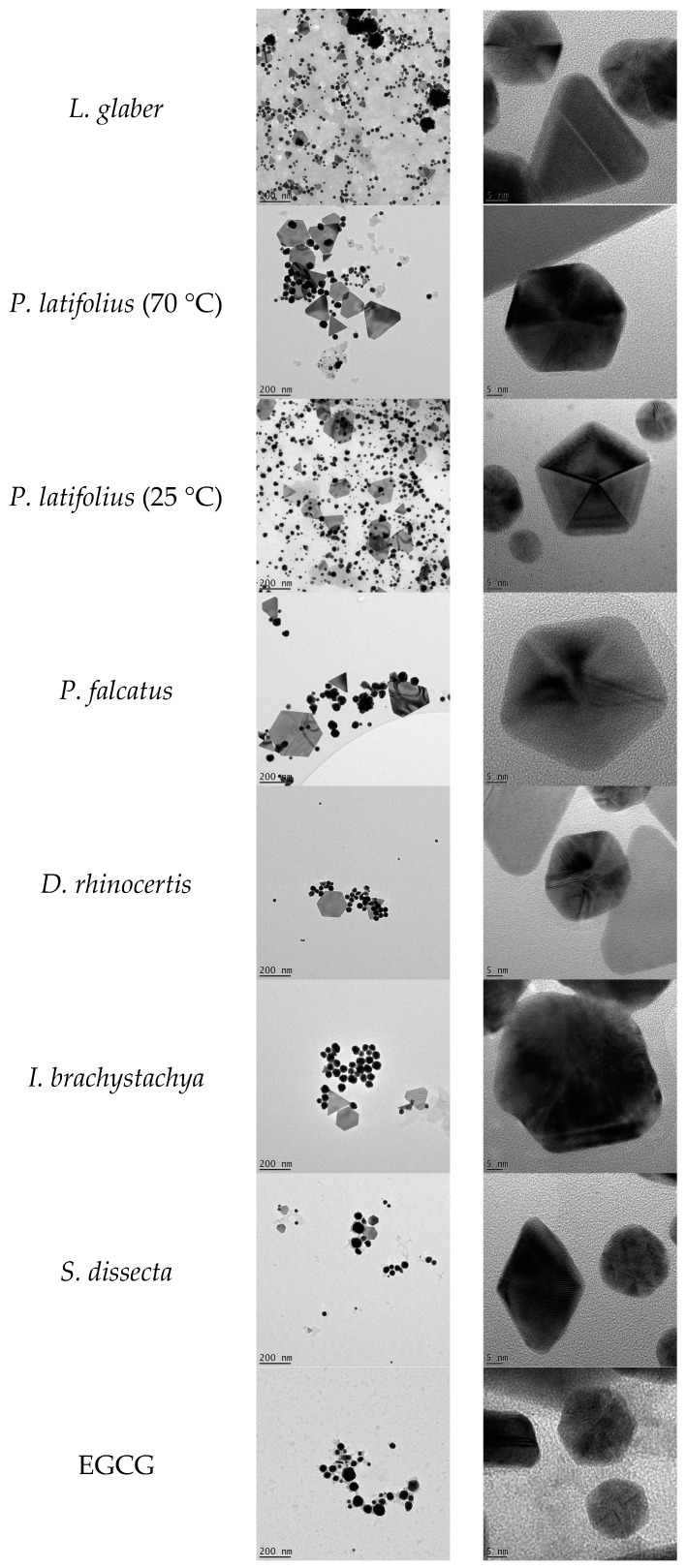
Transmission Electron Microscopy (TEM) images of the synthesized AuNPs.

The TEM images of the AuNPs, produced from plant extracts (Figure 2), show variable geometrical shapes and sizes. However, AuNPs produced from some extracts show a dominance of specific shapes over others. The presence of the anisotropic particles in the samples is indicated by the presence of absorbance towards the near infrared region in their spectra as discussed in Section 2.1. For instance, AuNPs produced with EGCG showed minimum absorbance in the near infrared region in the UV-Vis spectrum (Section 2.1) and TEM analysis (Figure 2) confirmed that these AuNPs were mostly uniform in shape. In contrast AuNPs synthesized from extracts produced from *S. dissecta* exhibited higher absorbance in the near infrared region in the UV-Vis spectrum (Sample 16 in Figure 1) and the TEM analysis shows that these AuNPs are more polydispersed (Figure 2).

**Figure 3:**
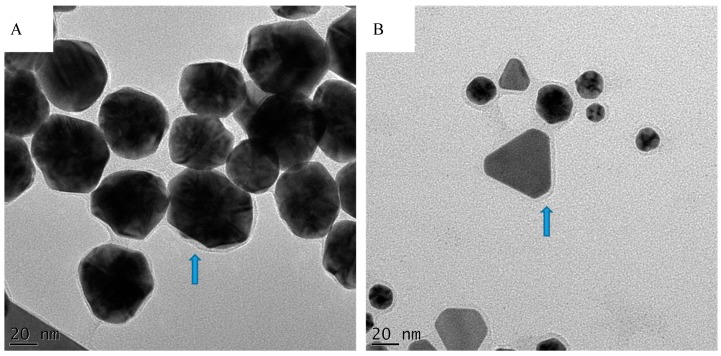
TEM images of AuNPs produced from (**A**) *I. brachystachya* and (**B**) *A. rubicundus*. The arrows point towards the halo surrounding the nanoparticles.

Overall, larger particles, from ~150 nm in size, were mostly triangular, truncated triangular, and hexagonal in shape. On the other hand, smaller nanoparticles were mostly, spherical, pentagonal and hexagonal, although a few small triangles could also be observed. This mixture of geometrical shapes is a common feature of AuNPs as reported before [32,33]. This is presumably due to the presence of cocktail of reducing phytochemicals in the extracts acting together to form the AuNPs. The fact that a singular phytochemical, EGCG, produced uniform AuNPs is supporting this assumption. Moreover, there was no apparent difference in the shapes between AuNPs synthesized at 25 °C and 70 °C (for the same plant extract) as seen for AuNPs synthesized from *P. latifolius* (Figure 2). One interesting observation from the TEM images is the presence of a halo surrounding most of the nanoparticles (Figure 3). This halo was also observed by Zeiri et al., which has a width of 2 to 3 nm, and was proposed that this halo the AuNPs from aggregation [34]. TEM images show only a few particles in each frame, hence statistically reliable distributions of these shapes and sizes cannot be evaluated using TEM analysis [34].

**Figure 4:**
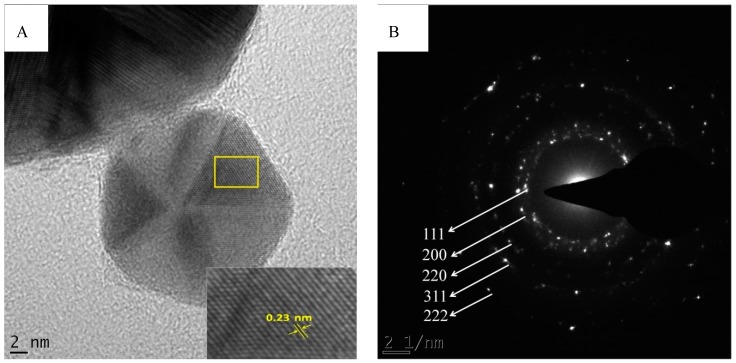
TEM images of *A. linearis* (rooibos tea) AuNPs showing (**A**) decahedron nanocrystal demonstrating the lattice fringes with spacing of 0.23 nm; (**B**) selected electron diffraction (SAED) pattern.

To illustrate the crystalline nature of the AuNPs, Figure 4A shows the fringe lattice of the AuNPs synthesized from *A. linearis* (rooibos tea). The fringe spacing was measured to be 0.23 nm, which closely matches the spacing between (111) plane of the face centred cubic (fcc) of gold (0.235 nm) [35]. Figure 4B shows the selected electron diffraction (SAED) pattern, which confirmed the crystalline nature of the AuNPs. The rings were indexed and was found to correspond to the (111), (200), (220), (311) and (222) reflections of fcc gold.

**Figure 5:**
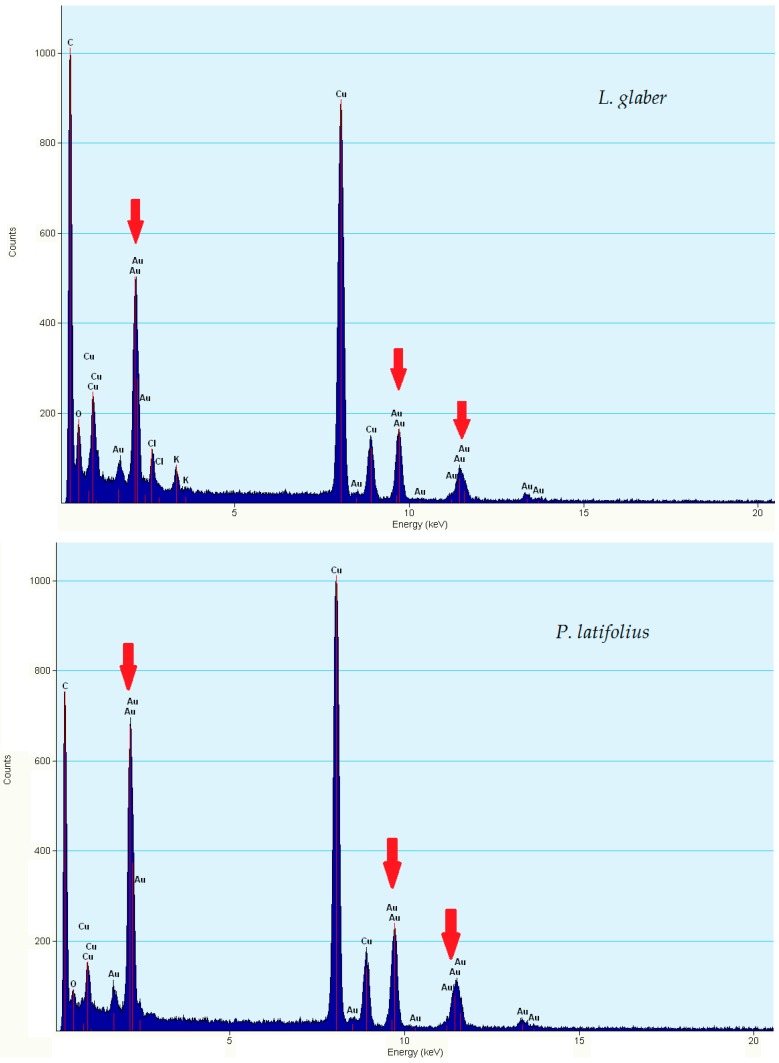
Energy Dispersive X-ray Spectroscopy (EDX) spectra of AuNPs from the extracts of *L. glaber* and *P. latifolius*. The red arrows show Au peaks.

The EDX spectroscopy analysis of the AuNPs confirmed the presence of gold ions in the samples selected for TEM analysis. Strong optical adsorption peaks were observed at around 2.3, 9.7 and 11.3 KeV (Figure 5), which are consistent with a previous study [20]. The presence of strong peaks of carbon, copper and silicon in some samples is attributed to the TEM grid and the detector window [36], whereas the presence of oxygen, potassium and chloride is suggested due to traces of the phytochemicals of the extracts and the gold salt [19,34].

### 2.3. Stability of the AuNPs

The zeta potential values of the synthesized AuNPs were measured in order to evaluate their stability. All measurements were done immediately after synthesis. Generally, the zeta potential measurements can be used to predict the long term stability of the AuNPs in a solution, as the magnitude of the charge is a reflection of the repulsion forces between the particles [37]. All of the plant extracts demonstrated negative zeta potential as shown in Table 1. A negative zeta potential value commonly suggests that the particles will be stable in solutions [37].

**Figure 6:**
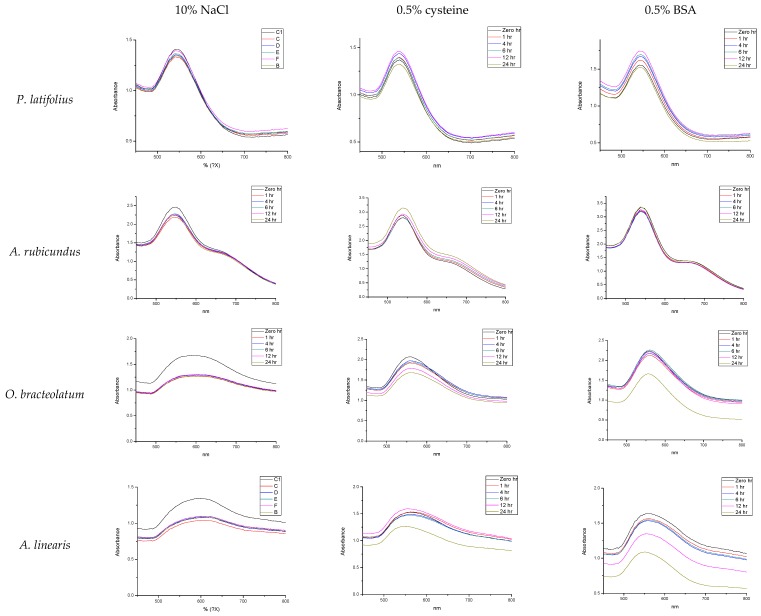
Changes in UV-Vis spectra of AuNPs over a 24-h period in buffers containing 10% NaCl, 0.5% cysteine and 0.5% Bovine Serum Albumin (BSA).

It is important that AuNPs, used in biological applications, retain their stability in biological environments. Biologically stable AuNPs are not expected to aggregate (manifested by minimal changes in their UV-Vis spectra) when placed, for extended periods, in buffers that simulate various biological conditions (e.g., salts and biological additives) [37]. In this study, the AuNPs were incubated with three different buffers solutions (10% NaCl, 0.5% cysteine and 0.5% Bovine Serum Albumin (BSA)). The stability of AuNPs was monitored by recording changes in UV-Vis spectra over time. Only AuNPs synthesized from *P. latifolius* and *A. rubicundus* demonstrated excellent stability by retaining their SPR (Figure 6). This indicates that these nanoparticles are highly stable at different environmental conditions and can therefore potentially be used in different biomedical applications. On the other hand, none of the other AuNPs synthesized from the other plant extracts showed a similar stability upon incubation with the same buffer solutions. For instance, the AuNPs generated from *O. bracteolatum* and *A. linearis* exhibited significant changes in their respective UV-Vis spectra as shown in Figure 6. It is suggested that the flattening in their SPR is due to the formation of larger particles. Also, the decrease of the intensities of the absorbance maxima may be due to the reduction of the AuNPs in terms of their number especially when incubated with 0.5% BSA.

### 2.4. Effect of Temperature on AuNPs Characteristics

**Figure 7:**
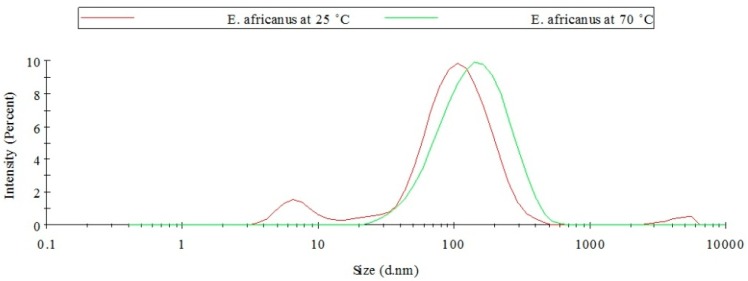
Particle size distribution for AuNPs produced from *E*. *africanus.*

In order to view the effect of elevated temperature on the overall synthesis of the AuNPs, the average values of the PD, λ_max_, and Pdi values were calculated (Table 2). It was previously reported that high temperatures could produce AuNPs of better size distribution [31]. The measurement of the particle size distribution of some AuNPs synthesized in this study, produced data that support that report. For example, Figure 7 shows that at a higher temperature the AuNPs synthesized from *E*. *africanus* exhibited better size distribution in contrast to the AuNPs synthesized at a lower temperature. Overall, particles synthesized at 70 °C were smaller and better defined, which is also correlated with the blue shift of the λ_max_. This is in agreement with the study conducted by Mountrichas and co-workers [31]. Another study reported by Song et al. explained that the formation of smaller AuNPs at higher reaction temperatures are due to the fact that gold ions are consumed in the formation of the nuclei with the increase of reaction temperature as a result of an increased reaction rate, which prevents the secondary reduction process of the formed nuclei and hence stops the formation of larger AuNPs [38].

### 2.5. Effect of Concentration and Determination of OC for Each Plant Extract

**Figure 8:**
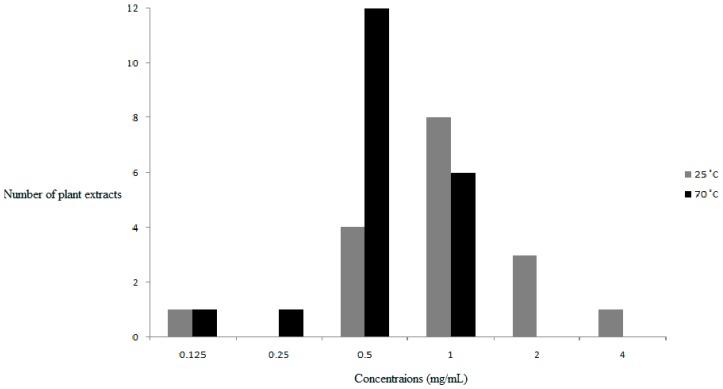
Optimum concentrations for AuNPs synthesis for all plant extracts at 25 °C and 70 °C.

In our study, the OC is considered the concentration of the plant extract at which the smallest and most uniform AuNPs are produced. To investigate the effect of plant extract concentration, fixed concentration of gold salt was incubated with different concentrations (16–0.007 mg/ml) of the plant extracts. The determination of the OC was firstly based on the SPR (λ_max_) and the uniformity of the UV-Vis curve, and secondly on the PD and Pdi values, which give an indication of the uniformity of the AuNPs produced. Synthesis to identify the OC was performed at a micro-scale in microtitre plates and thereafter the identified OCs were used to scale up the synthesis of the AuNPs to larger quantities. It was observed that higher temperatures yielded smaller particle sizes at lower concentrations of the plant extracts. Figure 8 shows that the OC at 25 °C is 1 mg/mL, while at 70 °C it is 0.5 mg/mL. No uniform AuNPs were synthesized at concentrations higher than 4 mg/mL.

**Figure 9:**
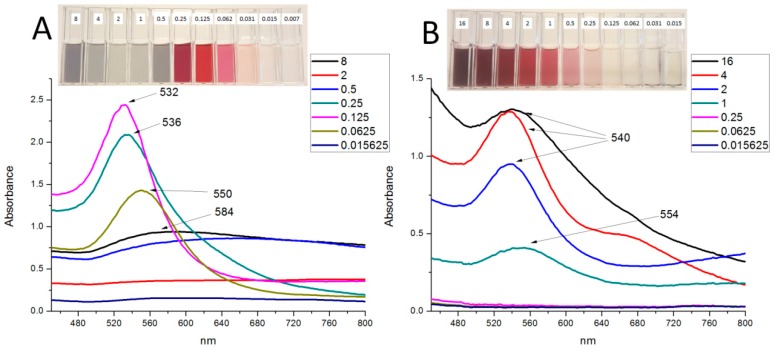
UV-Vis spectra for the AuNPs produced from (**A**) EGCG and (**B**) *P. latifolius* using different concentrations (mg/mL) at 25 °C. The insets show photos of the reaction mixture of different concentrations (mg/mL) of EGCG and *P. latifolius* after 1 h of reaction with gold salt.

In Figure 9, we show as an example, the changes in UV-Vis spectra as the concentrations of EGCG (one of the controls) and *P. latifolius* (one of the plant extracts) increase to demonstrate the growth pattern of the AuNPs at different concentrations. At low concentrations the SPR of both EGCG and *P. latifolius* exhibited red shifts, indicating the formation of AuNPs with a large particle size (at 0.0625 mg/mL EGCG produced λ_max_ of 550 nm with particles of an average diameter of 393 nm, while at 1 mg/mL *P. latifolius* produced λ_max_ of 554 nm with particles of an average diameter of 79 nm). By increasing the concentration of the reducing material (EGCG or the plant extracts) particles with smaller diameter was obtained and the OCs were reached. The SPR of the OCs exhibited the maximum blue shift compared to the SPR of the lower concentrations. At this point, the growth pattern for AuNPs of both EGCG and *P. latifolius* comes in agreement with the nucleation-growth mechanism of the citrate AuNPs proposed by Frens [39]. Frens showed that smaller citrate AuNPs can be obtained by increasing the sodium citrate (reducing agent) concentration while using fixed gold salt concentration. It was suggested that the citrate AuNPs were grown through a fast nucleation process followed by controlled diffusion growth at which average sizes can be reduced as sodium citrate concentrations increased. However, as we increase the concentration of the reducing material beyond the OC (0.125 mg/mL for EGCG and 2 mg/mL for *P. latifolius*) smaller AuNPs could not be obtained as expected. On the contrary, the SPR curves became more flat and/or red shifted as the concentrations increase, which is a clear indication of the increased average size of the AuNPs as discussed in Section 2.1. For instance, at a concentration of 0.25 mg/mL EGCG produced AuNPs with a diameter of 55 nm and a λ_max_ of 536 nm, while at a concentration of 0.125 mg/mL EGCG produced AuNPs with a diameter 45 nm and a λ_max_ 532 nm. In the case of *P. latifolius* no shifts were observed compared to the OC curve, but instead the SPR curves became more flat and showed absorbance above 600 nm, which are indications of the formation of larger particles (particle diameter of 83 nm at 4 mg/mL and 207 nm at 16 mg/mL).

Ji and co-workers [40], observed that citrate AuNPs were produced, with larger diameter, when increasing the sodium citrate concentration above a certain limit. They showed that two different pathways can produce AuNPs resulting in AuNPs with different sizes. These pathways were dependent on the pH of the reaction medium. They established that above a certain critical pH value, the AuNPs grow differently via Ostwald ripening, which leads to the formation of larger AuNPs. In their study, the pH of the reaction mixture was dependent on the initial sodium citrate/gold salt ratio. In another study, Guo and co-workers [41] also reported that the pH of the reaction medium affects the synthesis of AuNPs from *Eucommia ulmoides* bark aqueous extract. They showed that at pH values above a certain optimum value the reaction mixture turned blue due to the formation of larger and aggregated AuNPs according to the authors. This was also observed in our study with EGCG at 8 mg/mL (see inset Figure 9A). While the *P. latifolius* extract at the highest concentration tested (16 mg/mL) started to give blue/red colour compared to the rosy red colour obtained at the OC (see inset Figure 9B). We presume a similar mechanism occurs in our study as a change in pH was detected between the smallest and highest concentrations, even if it was small changes. 

We are also proposing other factor(s) that may as well be responsible for the non-linear relationship between the concentration of plant extracts and the formation of AuNPs. It is likely that, at concentrations above the OC the crowded reaction mixture causes a certain degree of impedance that prevent the reducing and/or capping agents to function effectively to produce AuNPs. The plant extracts generally contain a large number of phytochemicals. Some of these phytochemicals are present at high concentration and may insulate or even reverse the action of the reducing and/or capping agents. Therefore, the determination of OC at which the interaction of the plant extract with gold salt reach the optimum level is highly important and considered to be the optimal concentration for the synthesis of AuNPs, above the OC the growth pattern of the AuNPs change and produce larger particles.

### 2.6. Plant Phytochemicals Role in Bio-Reduction of Gold Salt

The bio-reduction of gold salt using plant extracts is mediated through the phytochemicals occluded within their aqueous extracts. These phytochemicals can mainly include free sugars (polysaccharides) and/or glycosidic containing derivatives (e.g., saponins), proteins, alkaloids and polyphenolic compounds (e.g., tannins and flavonoid derivatives), in addition to low percentage of lipophilic compounds coming to the solution by cosolvation. These phytochemicals provide polyfunctional matrix that has the ability to initiate the reduction of the gold ions and is also capable of providing stability to the AuNPs. Moreover, it appears that it is not just one type of phytochemical is responsible for the reducing actions of the plant extracts, but it can differ from one plant to another depending on the major phytochemical constituents in each plant. For instance, polyphenols and flavonoids were shown to be responsible for the formation of AuNPs from black tea [42]. AuNPs synthesized from *M. umbellatum* was attributed to the saponin content of the plant [20]. Organosulphur compounds were suggested to be the reducing agents in the synthesis of AuNPs from garlic (*Allium sativum*) [43]. AuNPs were also successfully synthesized from the aldehyde containing cinnamon essential oils [37]. The formation of AuNPs from *Cinnamomum camphora* was associated with the presence of terpenoids [44]. Dzimitrowicz and co-workers concluded that phenolic compounds play essential roles in the synthesis of AuNPs from some of *Lamiaceae* plants [45]. On the other hand, pure phytochemicals such as EGCG [23], apiin (apigenin-7-*O*-apioglucoside) [46], and guavanoic acid [47] were reported to have dual actions (as reducer and stabilizer) .

**Figure 10:**
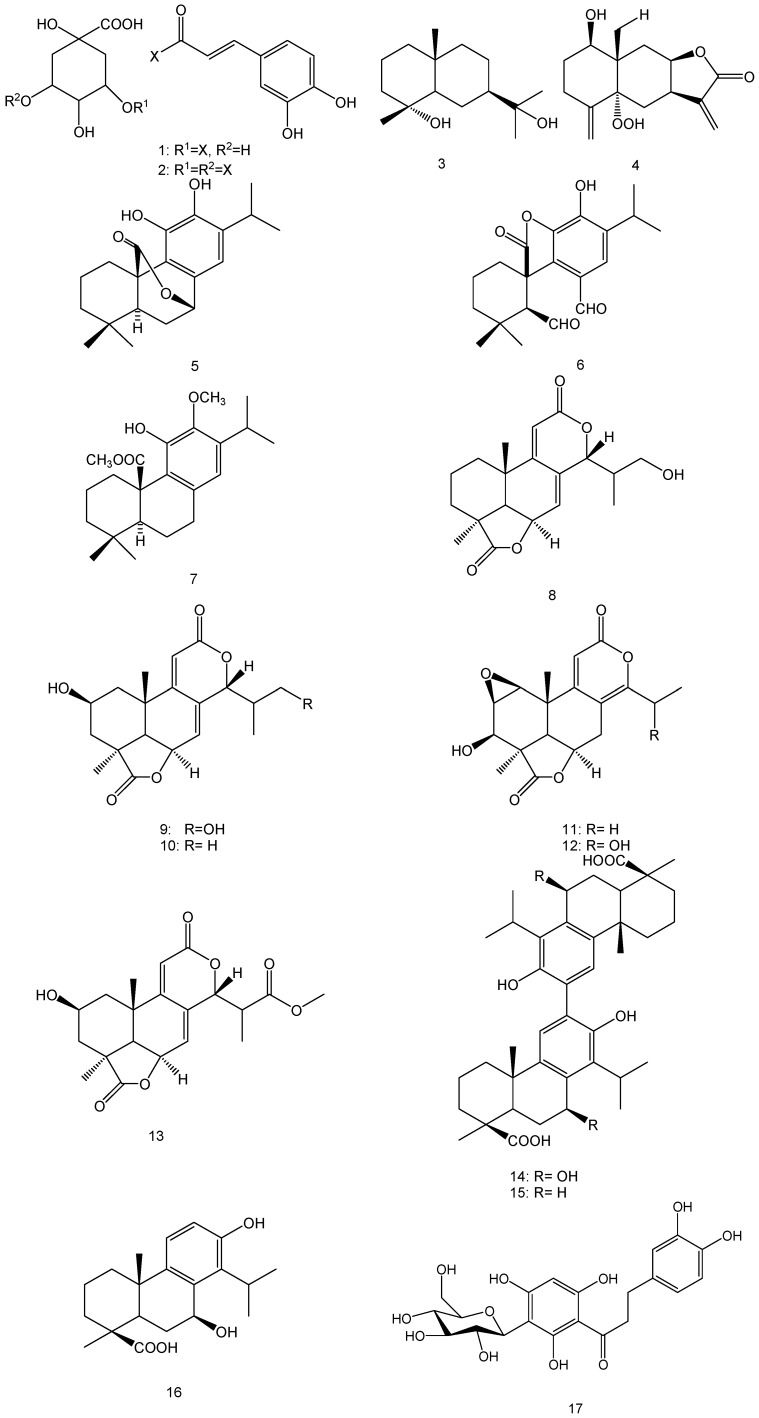
Chemical structures of compounds previously isolated from some of the plants used in this study.

Little is known about the chemistry of the tested plants in this study, yet some studies reported the isolation of various compounds that could possibly play a role in the formation of the AuNPs (Figure 10). For instance, several caffeic acid derivatives, e.g., 3-caffeoylquinic acid (**1**), 3,5-dicaffeoyluinic acid (**2**) were major phenolic compounds identified from the hydroethanolic extract of the aerial parts of *E. africanus* [48]. Additionally, a mixture of sesquiterpene lactones of the eudesmanolide type, e.g., 4α,11-dihydroxy-eudesmane (**3**) and 5α-hydroperoxy-eudesmane (**4**) were also reported [49]. Phenolic diterpenes namely; carnosol (**5**), rosmadial (**6**), and the 12-*O*-methyl-20-methyl ester derivative of carnosic acid (**7**) were identified from *S. africana-lutea* [50]. High phenolic contents were also observed in the aerial parts of *P. latifolius* and *P. falcatus*, with total flavonoid content ranging from 49.76 to 27.18 μg catechin/g dry sample [51]. A study of the chemical profile of the root extract of *P. falcatus* led to the isolation of several nagilactones *viz*; 16-hydroxynagilactone F (**8**), 2β,16-dihydroxynagilactone F (**9**), 2β-hydroxynagilactone F (**10**), nagilactone D (**11**), 15-hydroxynagilactone D (**12**) and nagilactone I (**13**), along with the bisditerpenoid; 7β-hydroxymacrophyllic acid (**14**), the totarol dimer macrophyllic acid (**15**) and the totarane-type diterpenoid inumakiol D (**16**) [52]. The flavone glycoside, Aspalathin (**17**), is a unique constituent of rooibos tea, which constitutes up to 9.3% of the dried leaves [53]. The chemical investigation of *C. africanum* led to the isolation of several pregnane glycosides named cynafoside C–H [54]. The aerial parts of *M. muricata* gave a mixture of unidentified triterpenes when studied chemically, while other *Metalisia* species afforded various chalcone derivatives [55]. Phytochemical investigation of several species of genus *Asparagus* led to isolation of several steroidal saponins [56,57,58].

The presence of certain part of structure like glucose unit(s) in some compound(s) in addition to phenolic or primary hydroxyls, peroxy, and aldehydic groups can play an important role in reducing gold salt. Also, the presence of carboxyl or hydroxyls groups in addition to the aromatic rings in different structural units can contribute to the stability of the AuNPs.

## 3. Materials and Methodology

### 3.1. Materials

*C. sinensis* (black and green tea) and *A. linearis* (rooibos tea) were purchased from local vendors in South Africa, EGCG from Zhejiang Yixin Pharmaceutical Co., Ltd. (Lanxi City, China), 96 well polystyrene microplates from Greiner bio-one GmbH (Frickenhausen, Germany), gold salt (sodium tetrachloroaurate (III) dehydrate) from Sigma-Aldrich (Cape Town, South Africa), Bovine Serum Albumin (BSA) from Miles Laboratories (Pittsburgh, Pa, USA), *N*-Acetyl-l-cysteine from Boehringer Mannheim GmbH (Mannheim, Germany) and sodium chloride (NaCl) from Merck (Cape Town, South Africa).

### 3.2. Instruments

Centrifugation for the extracts was done using Allegra^®^ X-12R (Beckman Coulter, Cape Town, South Africa). The AuNPs were centrifuged using Centrifuge 5417R (Eppendorf AG, Hamburg, Germany). The extracts were freeze dried using FreeZone 2.5 L (Labconco, Kansas City, MO, USA). UV-Vis spectra were recorded using POLARstar Omega microplate reader (BMG Labtech, Cape Town, South Africa). The particle size, size distribution and zeta potential measurements of the freshly synthesized AuNPs in solution were analysed using Zeta sizer (Malvern Instruments Ltd., Malvern, UK). TEM analysis was done using FEI Tecnai G^2^ 20 field-emission gun (FEG).

### 3.3. Plant Collection

Aerial plant samples (Table 3) were collected during May 2015 from two sites in the Western Cape Province of South Africa. The first site is situated in Malmesbury (GPS coordinates: 33°27′33.44″S, 18°44′39.87″E) approximately 60 km north of the University of the Western Cape. The vegetation on this site is classified as the critically endangered Swartland Granite Renosterveld. The second collection site is situated approximated 20 km south of the University of the Western Cape in Mfuleni (GPS coordinates: 34°00′18.54″S, 18°41′8.19″E) that supports an endangered vegetation unit known as Cape Flats Dune Strandveld. On both sites the sampling strategy was random and all the specimens collected were identified and deposited at the Compton Herbarium (NBG), Kirstenbosch, Cape Town, South Africa. The collection and identification process was performed by C.N. Cupido the co-author of this paper.

### 3.4. Preparation of the Plant Extracts

Fresh plant materials were dried in the shade for two weeks. After drying, the plant materials were grinded and extracted using boiled distilled water (50 mL of distilled water added to 5 g of each plant powder). The plant decoctions were then centrifuged at 3750 rpm for 2 h. The supernatants were then freeze dried. A stock solution of 32 mg/mL was freshly prepared for each extract before the screening step.

### 3.5. Screening of Gold Nanoparticles Synthesis

In a 96 well plate, 250 μL of 1 mM [20] gold salt were added to 50 μL of plant extracts stock solutions with increasing concentrations (0.007 to 16 mg/mL). The plates were incubated at 25 °C and 70 °C with shaking (40 rpm). After 1 h of shaking, the SPR of the AuNPs was measured by recording the UV-Vis spectrum ranging from 300 nm to 800 nm. For further characterization and stability evaluations, the synthesis of the AuNPs from the tested plant extracts was scaled up using the OC of the plant extracts.

### 3.6. High Resolution Transmission Electron Microscopy (HRTEM) and Energy Dispersive X-ray Spectroscopy (EDX) Analysis

To study the surface morphology of the AuNPs, samples were prepared by drop-coating one drop of each sample solution onto a holey carbon coated copper grid. This was then dried under a Xenon lamp for 10 min, where after the sample coated grids were analysed under the microscope. Transmission electron micrographs were operated in bright field mode at an accelerating voltage of 200 kV. Energy dispersive X-ray spectra were collected using an EDAX liquid nitrogen cooled Lithium doped Silicon detector.

### 3.7. Stability Testing of the Synthesized AuNPs

The in vitro stability of the synthesized AuNPs was measured by incubating the AuNPs with three aqueous buffer solutions (NaCl, cysteine and BSA). First, the synthesized AuNPs were centrifuged at 10,000 rpm for 5 min. The pellets were washed three times with distilled water to remove phytochemicals that are not capping the AuNPs. The nanoparticles were re-suspended in 1 mL autoclaved distilled water. Thereafter, 100 µL of the tested AuNPs solutions was incubated with equal volume of the buffer solutions in a 96 well plate. The final concentrations of the biological media in the final mixture were as follow; 10% NaCl, 0.5% cysteine and 0.5% BSA. The stability of the AuNPs was evaluated by measuring the changes in UV-Vis spectra after 1, 4, 6, 12 and 24 h.

## 4. Conclusions

It is well known that plants contain countless numbers of primary and secondary metabolites and we therefore expected that characteristically different AuNPs can by synthesized using aqueous extracts of different plants. Since the phytochemical constituent in one plant extract is different from another, the use of different plant extracts can lead to dissimilar degree of bio-reduction of gold salt to synthesize AuNPs of variable shape, size, dispersity and bio-stability. This variability may also extend their potential in biological activities and other applications. This study shows for the first time a quick and easy screening method of a large number of aqueous plant extracts for the bio-synthesis of AuNPs on micro-scale level. Considering the number of plant species that can potentially be screened, this approach has several advantages since it reduces cost and time of the synthesis. In this study we also investigated the effect of the concentration of different plant extracts on the formation of AuNPs. The results showed a non-linear relationship between the concentrations of the plant extracts and the formation of AuNPs. We determined an optimum concentration for each plant extract at which the smallest and most uniform AuNPs were obtained. The synthesized AuNPs were thoroughly characterised, and their shapes, size and in vitro stability were examined. The formation of the AuNPs was confirmed by the presence of characteristic SPR in the UV-Vis spectra. We applied different reaction conditions to study the effect of temperature on the synthesis and showed that smaller particle size and more defined AuNPs can be synthesized at a higher temperature. The particle sizes were studied by DLS analysis, which also gave an indication about the stability of AuNPs in aqueous solutions. In agreement with the UV-Vis data, TEM analysis showed that AuNPs of various geometrical shapes were produced. EDX analysis also confirmed the presence of gold ions in the samples. By monitoring changes in the SPR bands of the synthesized AuNPs when incubated with different buffer solutions, we were able to determine that AuNPs produced form two plant extracts namely, *P. latifolius* and *A. rubicundus* are highly stable in these buffers which suggested that these AuNPs will be very stable in biological environments. These AuNPs may be suitable for various biomedical applications such as drug delivery.

## Figures and Tables

**Table 1:** The optimum concentration (OC), particle diameter (PD), the polydispersity index (Pdi), λ_max_ and average zeta potential values (ZP) of the AuNPs synthesized from the plant extracts at 25 °C and 70 °C.

Plant name	25 °C					70 °C				
	OC (mg/mL)	λ_max_ (nm)	PD (nm)	Pdi	ZP (mV)	OC (mg/mL)	λ_max_ (nm)	PD (nm)	Pdi	ZP (mV)
1 *Aspalathus hispida*	*	*	*	*	*	0.5	534	34	0.564	−13
2 *Aspalathus linearis*	0.5	536	99	0.343	−23	0.5	536	61	0.315	−20
3 *Asparagus rubicundus*	*	*	*	*	*	1	538	28	0.66	−22
4 *Cynanchum africanum*	2	558	110	0.396	−14	0.5	546	99	0.407	−21
5 *Dicerothamnus rhinocertis*	*	*	*	*	*	0.5	534	63	0.551	−41
6 *Eriocephalus africanus*	1	554	67	0.565	−23	0.5	542	102	0.326	−23
7 *Hermannia alnifolia*	1	536	78	0.398	−21	0.5	536	66	0.42	−27
8 *Indigofera brachystachya*	1	548	87	0.371	−16	1	534	100	0.477	−41
9 *Lobostemon glaber*	0.5	552	218	0.76	−23	0.5	540	136	0.217	−26
10 *Metalasia muricata*	1	546	65	0.469	−16	0.25	544	61	0.352	−14
11 *Nidorella foetida*	0.5	564	124	0.231	−24	0.5	548	97	0.243	−28
12 *Otholobium bracteolatum*	4	546	47	0.525	−20	1	542	53	0.423	−25
13 *Podocarpus falcatus*	1	538	141	0.6	−15	0.5	540	102	0.577	−15
14 *Podocarpus latifolius*	2	540	76	0.46	−16	1	540	54	0.513	−18
15 *Salvia africana-lutea*	1	534	148	0.466	−25	0.5	534	69	0.202	−23
16 *Searsia dissecta*	0.5	538	62	0.405	−12	0.5	538	68	0.299	−14
17 *Senecio pubigerus*	1	538	75	0.519	−18	0.5	536	49	0.469	−15
18 *Camellia sinensis* (Black tea)	2	538	63	0.535	−0.2	1	540	23	0.67	−19
19 *Camellia sinensis* (Green tea)	1	535	104	0.61	−12	1	534	47	0.376	−12
20 EGCG	0.125	532	45	0.324	−26	0.125	534	52	0.311	−22

* No nanoparticles were synthesized at this condition.

**Table 2:** The average PD, λ_max,_ and polydispersity index (Pdi) values obtained from all the plant extracts at 25 °C and 70 °C.

Nanoparticles Characteristics	25 °C	70 °C
λ_max_ (nm)	543 ± 9	539 ± 4
particle diameter (nm)	97 ± 44	68 ± 29
Pdi	0.465 ± 0.127	0.419 ± 0.14

**Table 3:** The collected plants and their accession numbers.

Plant Name	Family Name	Accession Number
*Aspalathus hispida*	Fabaceae	1463158/NBG
*Asparagus rubicundus*	Asparagaceae	1463146/NBG
*Cynanchum africanum*	Apocynaceae	1463157/NBG
*Dicerothamnus rhinocertis*	Asteraceae	1463148/NBG
*Eriocephalus africanus*	Asteraceae	1463147/NBG
*Hermannia alnifolia*	Malvaceae	1463145/NBG
*Indigofera brachystachya*	Fabaceae	1463156/NBG
*Lobostemon glaber*	Boraginaceae	1463149/NBG
*Metalasia muricata*	Asteraceae	1463150/NBG
*Nidorella foetida*	Asteraceae	1463153/NBG
*Otholobium bracteolatum*	Fabaceae	1463155/NBG
*Podocarpus falcatus*	Podocarpaceae	*
*Podocarpus latifolius*	Podocarpaceae	*
*Salvia africana-lutea*	Lamiaceae	1463154/NBG
*Searsia dissecta*	Anacardiaceae	1463151/NBG
*Senecio pubigerus*	Asteraceae	1463152/NBG

* *P. falcatus* and *P. latifolius* were purchased from Kirstenbosch national botanical garden, Cape Town, South Africa.
